# Developmental lung expression and transcriptional regulation of Claudin-6 by TTF-1, Gata-6, and FoxA2

**DOI:** 10.1186/1465-9921-15-70

**Published:** 2014-06-26

**Authors:** Felix R Jimenez, Joshua B Lewis, Samuel T Belgique, Tyler T Wood, Paul R Reynolds

**Affiliations:** 1 Department of Physiology and Developmental Biology, Brigham Young University, 3054 Life Sciences Building, Provo, UT 84602, USA

**Keywords:** Lung, Claudin-6, Transcription, Immunofluorescence

## Abstract

**Background:**

Claudins are transmembrane proteins expressed in tight junctions that prevent paracellular transport of extracellular fluid and a variety of other substances. However, the expression profile of Claudin-6 (Cldn6) in the developing lung has not been characterized.

**Methods and results:**

Cldn6 expression was determined during important periods of lung organogenesis by microarray analysis, qPCR and immunofluorescence. Expression patterns were confirmed to peak at E12.5 and diminish as lung development progressed. Immunofluorescence revealed that Cldn6 was detected in cells that also express TTF-1 and FoxA2, two critical transcriptional regulators of pulmonary branching morphogenesis. Cldn6 was also observed in cells that express Sox2 and Sox9, factors that influence cell differentiation in the proximal and distal lung, respectively. In order to assess transcriptional control of *Cldn6*, 0.5, 1.0, and 2.0-kb of the proximal murine *Cldn6* promoter was ligated into a luciferase reporter and co-transfected with expression vectors for TTF-1 or two of its important transcriptional co-regulators, FoxA2 and Gata-6. In almost every instance, TTF-1, FoxA2, and Gata-6 activated gene transcription in cell lines characteristic of proximal airway epithelium (Beas2B) and distal alveolar epithelium (A-549).

**Conclusions:**

These data revealed for the first time that Cldn6 might be an important tight junctional component expressed by pulmonary epithelium during lung organogenesis. Furthermore, Cldn6-mediated aspects of cell differentiation may describe mechanisms of lung perturbation coincident with impaired cell junctions and abnormal membrane permeability.

## Background

Lung development is a complex and coordinated process that requires cellular differentiation and interaction between respiratory epithelial cells and the surrounding mesenchymal environment [1]. As lung development proceeds, compartmentalization is orchestrated in large part by tight junctions (TJs) between neighboring epithelial cells [2]. Accordingly, developing pulmonary epithelium obtains a polarized conformation and it refines mechanisms that regulate ion and molecular transport between apical and basolateral compartments [3]. TJs are protein complexes comprising several components including transmembrane proteins such as Claudins (Cldn), Occludins, and junctional adhesion molecules (JAMs) stabilized by various cytoplasmic and cytoskeletal proteins [4-6].

The Claudin (Cldn) family of proteins plays a critical role in TJs by establishing a junction complex (cellular pore) that controls extracellular ion movement at cell-cell apposition [7,8]. The Cldn family is comprised of 27 highly conserved membrane proteins that, similar to other tetraspanins, traverse the cellular membrane four times and contain two EL loops where interdigital interactions with other Cldns occur [9,10]. While Cldn expression is both temporally and spatially controlled, altered expression of Cldn members may contribute to the modification of intracellular permeability and molecular transport selectivity by specific epithelial cell types [8,11].

Among Cldn proteins, Claudin-6 (Cldn6) plays a fundamental role in epithelial differentiation and permeability. Embryonic expression of Cldn6 has been identified during epidermal morphogenesis and is critical for epidermal differentiation and epithelial barrier formation [12]. For instance, specific studies have shown that sufficient expression of Cldn6 correlated with the establishment of the permeability barrier’s integrity and function, and overexpression of Cldn6 is associated with defects in epidermal permeability [13]. Complimentary studies employing the overexpression of Cldn6 in transgenic mice resulted in lethal epidermis-related defects including poor temperature stabilization, infection by microorganisms through the skin, and uncontrolled water loss [9,14]. While there is a general consensus that Cldn6 participates in TJs that maintain homeostasis, abnormalities in its expression or function may also lead to tumorigenesis. For example, Cldn6 expressed by mammary epithelial cells functions as a tumor suppressor [15-17] and its downregulation has been implicated in neoplasticity and metastatic disease development [16,18].

In the current study, we assessed the precise temporal and spatial distribution of Cldn6 in the embryonic mouse lung. Through immunofluorescent assessment, we discovered precise expression of Cldn6 and co-expression with the critical pulmonary transcription factors thyroid transcription factor 1 (TTF-1), forkhead box A2 (FoxA2), and Gata-6 in Cldn6 expressing cells. Additional experiments that test the hypotheses that these same factors transcriptionally regulate *Cldn6* were also performed. Collectively, data presented suggest controlled pulmonary Cldn6 expression and the likelihood that Cldn6 functions in distinct developmental roles. While such roles have remained largely undiscerned to this point, ongoing research may clarify important Cldn6 functions in differentiating pulmonary epithelium.

## Methods

### Mice

C57Bl/6 mice were housed and maintained in a conventional animal facility in accordance with institutional guidelines and approved Institutional Animal Care and Use Committee (IACUC) protocols. Embryonic mice were obtained from dams on the days specified following the formation of a vaginal plug that identified embryonic (E) day 0 [19].

### Antibodies and Immunofluorescence

An anti-Cldn6 goat polyclonal antibody (C-20, Santa Cruz Biotechnologies, Santa Cruz, CA) was used at a dilution of 1:20 to identify Cldn6 expression in lung cells at different stages of lung development from E11.5 to post natal day (PN) 1. Co-localizing experiments were also conducted with the following antibodies: TTF-1 (1:100 from Seven Hills BioReagents, Cincinnati, OH), FoxA2 (1:100 from Seven Hills BioReagents), Sox2 (1:100 from Seven Hills BioReagents), and Sox9 (1:100 from Santa Cruz Biotechnologies).

Immunofluorescent staining for Cldn6, TTF-1, FoxA2, Sox2, and Sox9, were performed using standard techniques. Briefly, 5-μm paraffin sections from E11.5 to PN1 were deparaffinized and rehydrated by incubation in decreasing ethanol concentrations. Antigen retrieval was then performed as already outlined [20]. Following antigen retrieval, sections were blocked in 5% donkey serum in PBS for 2 hours at room temperature, followed by incubation with primary antibodies at 4°C overnight. Control sections were incubated in blocking serum alone. After overnight incubation, all sections (including the controls) were washed using PBS/triton prior to the application of fluorescent-conjugated secondary antibodies for 1 hour at room temperature. Specifically, Alexa Fluor® 488 Rabbit Anti-Goat IgG was used for Cldn6 and Alexa Fluor 633 goat anti-rabbit IgG secondary antibodies were used against all other primaries (Invitrogen, Carlsbad, CA). For dual label immunofluorescence, secondary antibodies were initially applied to the strongest primary in order to minimize decreasing intensity resulting from subsequent washes. All sections were mounted using VectaShield containing DAPI (Vector Laboratories, Burlingame, CA).

### Microarray analysis and qRT-PCR

Microarray experiments were designed and mRNA analysis was conducted as already described [21]. In order to specifically assess *Cldn6* mRNA expression throughout development, total RNA was isolated from whole mouse lungs at various time points with an Absolutely RNA RT-PCR Miniprep Kit (Stratagene, La Jolla, CA) and treated with DNase. Reverse transcription of RNA was performed using the Invitrogen Superscript III First-Strand Synthesis System (Life Technologies, Grand Island, NY) in order to obtain cDNA for PCR. The following primers were synthesized and HPLC purified by Invitrogen Life Technologies: Cldn6 (For-GCA GTC TCT TTT GCA GGC TC and Rev-CCC AAG ATT TGC AGA CCA GT) and GAPDH (For-TAT GTC GTG GAG TCT ACT GGT and Rev-GAG TTG TCA TAT TTC TCG TGG). cDNA amplification and data analysis were performed using Bio Rad iQ SYBR Green Supermix (Bio-Rad Laboratories, Hercules, CA) and a Bio Rad Single Color Real Time PCR detection system (Bio-Rad Laboratories). Primers were used at a concentration of 75 nM each in 25-μl reactions. Cycle parameters were as follows: 3 min at 95°C for initial denaturation, followed by 40 cycles composed of 1 min at 95°C, 15 sec at 55°C and 15 s at 72°C. Control wells lacking template or RT were included to identify primer-dimer products and to exclude possible contaminants.

### Plasmid construction and reporter gene assays

0.5-kb, 1.0-kb and 2.0-kb of the proximal mouse *Cldn6* promoter were obtained by polymerase chain reaction (PCR) using the Expand High Fidelity PCR System (Roche, Indianapolis, IN). The amplified *Cldn6* promoter fragments were cloned and directionally ligated into the pGL4.10-basic luciferase reporter plasmid (Promega, Madison, WI) and verified by sequencing as described previously [22].

Functional assays of reporter gene constructs were performed by transient transfection of Beas2B and A-549 cells (American Tissue Culture Collection, ATCC, Manassas, VA) using FuGENE-6 HD reagent (Promega) [23]. Beas2B is a transformed human bronchiolar epithelial cell line and A-549 is a human pulmonary adenocarcinoma cell line characteristic of alveolar type II cells [24]. Transfections included 100 ng of pGL4.10-0.5 kb-Cldn6, pGL4.10-1.0 kb-Cldn6 or pGL4.10-2.0 kb-Cldn6, 100 ng pRSV-βgal in order to assess transfection efficiency, and 100 ng of expression vectors for key transcription factors including TTF1 (pCMV-TTF-1), FoxA2 (pCMV-FoxA2), or Gata-6 (pCMV-Gata-6). In the place of expression vectors, 100 ng of pcDNA control vector was added to equilibrate total DNA concentration at 300 ng. After 48 hours, plates were scraped and centrifuged, and the cleared supernatant was screened for total enzymatic β-gal expression to evaluate efficiency and luciferase activity [25]. Luciferase activity was determined in 20 μl of extract at room temperature with 80 μl of luciferase substrates (Promega, Madison, WI) for 10 sec after a 2-sec delay in a Moonlight™ 3010 luminometer (BD Biosciences, San Jose, CA).

### Statistical analysis

Reporter values are expressed as mean ± SD obtained from at least three separate experiments in each group. Data were assessed by one- or two-way analysis of variance (ANOVA). When ANOVA indicated significant differences, the Student *t*-test was used with Bonferroni correction for multiple comparisons. Results presented are representative, and those with P values < 0.05 were considered significant. Messenger RNA microarray data was normalized using the Robust Multichip Average model and analyzed using three different statistical methods, including Bayesian Analysis of Time Series (BATS), Extraction of Differential Gene Expression (EDGE), and two-way ANOVA [21].

## Results

### Cldn6 expression during mid to late prenatal lung development

**Figure 1:**
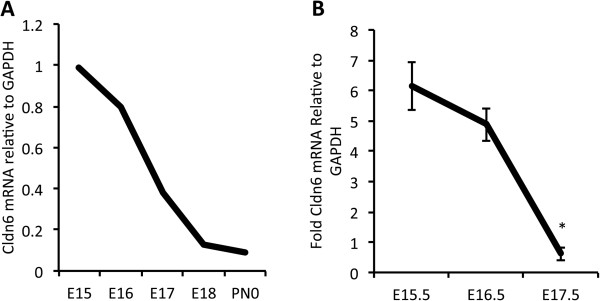
**Cldn6 mRNA Expression**. Control C57Bl6 mice were screened by microarray analysis and *Cldn6* expression levels were derived relative to GAPDH from E15-PN0 **(A)**. Confirmatory quantitative RT-PCR was conducted using total RNA from embryonic C57Bl6 mice and results are presented relative to GAPDH **(B)**. Representative data from experiments performed in triplicate are shown. *P ≤ 0.05 when comparisons were made between E15.5 and E16.5 or E15.5 and E17.5.

**Figure 2:**
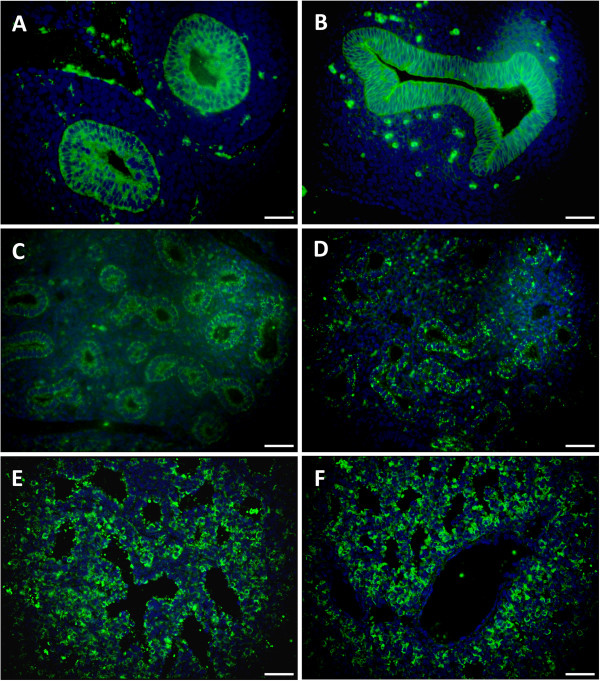
**Cldn6 was immunolocalized during periods of murine lung morphogenesis.** Cldn6 was initially detected in primitive respiratory epithelium at E12.5 **(A)** and expression persisted in differentiating epithelial cells at E13.5 **(B)**, E14.4 **(C)**, E15.5 **(D)**, E16.5 **(E)**, and E17.5 **(F)**; however, expression diminished as development proceeded. Cldn6 immunoflorescence was revealed by Alexa Fluor® 488 secondary antibodies and DAPI staining was performed for cellular perspective. No immunoreactivity was observed in lung sections incubated without primary antibodies (not shown) and all images are at 40X original magnification. Scale bars represent 20 μm.

*Cldn6* mRNA was initially evaluated by mRNA microarray analysis throughout a developmental time course in order to determine changes in its expression during periods of lung development and maturation. Lung samples from C57Bl6 mice were obtained daily from E12 through PN0 and were hybridized to the Mouse Gene 1.0ST Array (n = 3 per time point). Dynamic changes in the expression of *Cldn6* were detected as development progressed and the data suggested a precipitous decline in expression from E15 to PN0 (Figure 1A). Quantitative RT-PCR of total C57Bl6 lung RNA was used to validate the expression profile of *Cldn6* over the same period. There was a strong correlation between mRNA expression detected by microarray analysis (Figure 1A) and assessment by quantitative RT-PCR despite notable differences in magnitude (Figure 1B).Immunofluorescence was next used in order to correlate protein availability and localization with mRNA expression identified by microarray analysis and quantitative RT-PCR. Expression of Cldn6 was pronounced in the primitive pulmonary tubules of the developing lung from E12.5-E13.5 and the expression was intensely specific to resident epithelial cells (Figure 2A and C). In particular, Cldn6 was detected at not only the apical domain classically associated with tight junctions, but along the basolateral areas as well. The detection of fluorescence diminished as development continued; however, notable expression of Cldn6 was still detected in the primitive conducting and respiratory tubules from E14.5 through E17.5 (Figure 2E,G,I, and K).

### Cldn6 expression coincided with TTF-1 expressing primitive pulmonary epithelium

**Figure 3:**
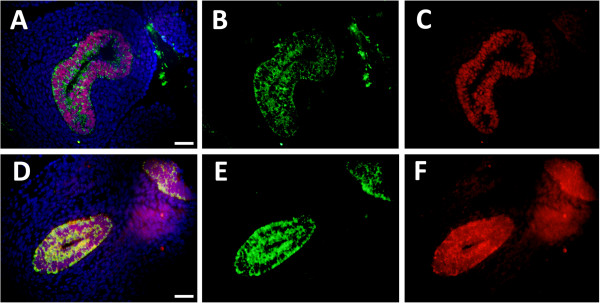
**Cldn6 was co-localized with TTF-1 at E12.5 (A-C) and E13.5 (D-F).** Merged images are shown (**A** and **D**) that include Cldn6 immunofluorescence (**B** and **F**), TTF-1 immunofluorescence (**C** and **F**) and DAPI staining for cellular perspective. No immunoreactivity was observed in lung sections incubated without primary antibodies (not shown) and all images are at 40X original magnification. Scale bars represent 20 μm.

Thyroid transcription factor (TTF)-1 is a member of the Nkx2 transcription factor family of homeodomain-containing proteins expressed by the lung, thyroid, ventral forebrain, and pituitary [26]. TTF-1 has been consistently implicated in lung development and its functions include the activation of critical gene programs that control pulmonary epithelial cell differentiation during lung morphogenesis [27]. Because Cldn6 expression was localized to developing pulmonary epithelium, we sought to determine whether Cldn6 and TTF-1 were co-expressed. Co-immunofluorescence revealed that Cldn-6 and TTF-1 expression were both detected in developing epithelial cells at E12.5 and E13.5 (Figure 3A and E). Although TTF-1 expression was nuclear and Cldn6 remained primarily localized to the plasma membrane, concomitant expression was clear through E13.5. While expression patterns continued to overlap, co-expression diminished through PN1 (not shown).

**Figure 4:**
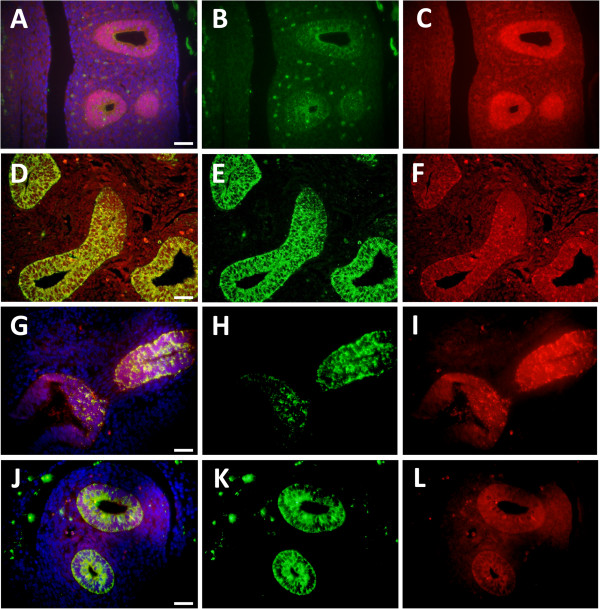
**At E13.5, Cldn6 immunofluorescence was co-localized with FoxA2 (A-C), Gata-6 (D-F), Sox2 (G-I), and Sox9 (J-L).** Merged images are shown **(A, D, G and J)** that include Cldn6 **(B, E, H, and K)**, and FoxA2 **(C)**, Gata-6 **(F)**, Sox2 **(I)**, or Sox9 **(L)**. DAPI staining was performed for cellular perspective. No immunoreactivity was observed in lung sections incubated without primary antibodies (not shown) and all images are at 40X original magnification. Scale bars represent 20 μm.

Because Cldn6 expression coincided with the manifestation of TTF-1, we next sought to determine whether Forkhead box A2 (FoxA2) and Gata-6 were observed in Cldn6-expressing cells. FoxA2 is an important nuclear lung transcription factor that contains a winged helix DNA binding domain and Gata-6 is a zinc finger factor that critically influences endoderm formation via the activation of target genes [28]. FoxA2 is required for foregut formation during early periods of embryogenesis [29] and it often partners with TTF-1 in the genetic orchestration of respiratory epithelial cell differentiation [30]. Despite weaker co-expression, membranous Cldn6 expression overlapped with FoxA2 at E13.5 (Figure 4A). Gata-6 expression at E13.5 was also highly consistent with the expression of Cldn6 (Figure 4D).

### Cldn6 was observed in Sox2- and Sox9-expressing cells

The molecular phenotypes of developing proximal and distal lung epithelial cell lineages have been associated with the differential expression of the transcription factors Sox2 and Sox9—sex-determining region Y (SRY)-box 2 and 9 [31]. Sox genes are highly conserved throughout the animal kingdom [32] and Sox2 has been implicated as an early marker for proximal lung cell differentiation [33] whereas Sox9 has been increasingly connected with distal respiratory trajectories [34]. Due to plausible contributions to lung cell delineation, Cldn6 immunofluorescence was used to test whether Cldn6 was expressed with Sox2 and Sox9 during early periods of lung development. Our data demonstrated that Sox2 and Sox9 were both co-expressed with Cldn6 in developing pulmonary epithelium at E12.5 (not shown) and E13.5 (Figure 4G and J).

### TTF-1, FoxA2, and Gata-6 transcriptionally regulated Cldn6

**Figure 5:**
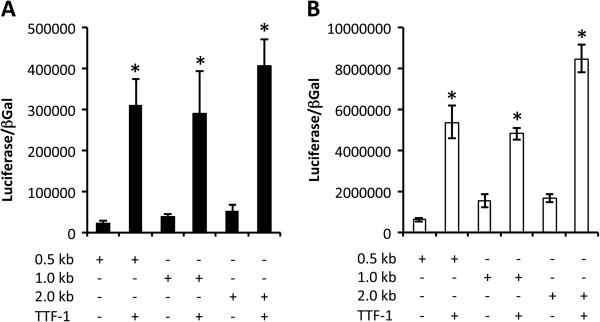
**TTF-1 induced *Cldn6 *transcription in bronchiolar Beas2B cells (A) and A-549 alveolar type II-like epithelial cells (B).** TTF-1 induced transcription by acting on the 0.5-kb, 1.0-kb, and 2.0-kb proximal mouse *Cldn6* promoters ligated into luciferase reporter vectors. In each case, TTF-1 significantly induced the transcription of the *Cldn6* gene. Significant differences in luciferase activity, normalized to β-galactosidase used to assess transfection efficiency, are noted at *P ≤ 0.05 when compared to non TTF-1 transfected cells. The data shown are representative of experiments performed in triplicate.

**Figure 6:**
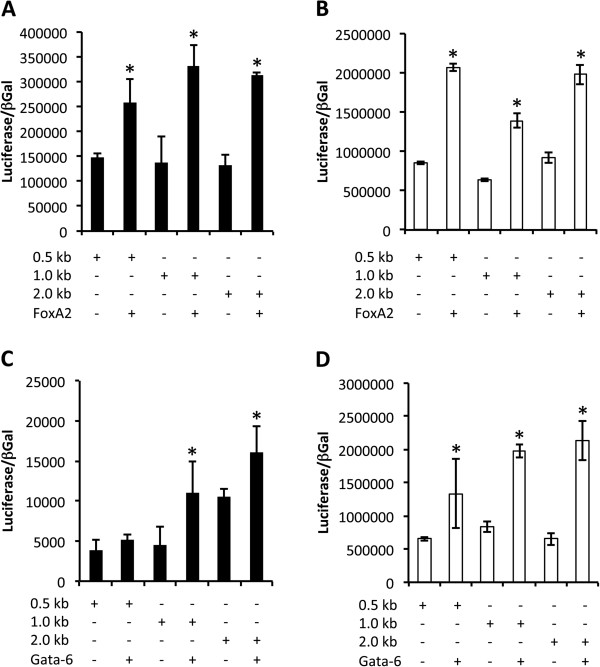
**FoxA2 (A and B) and Gata-6 (C and D) induced *Cldn6 *transcription in bronchiolar Beas2B cells (A and C) and A-549 alveolar type II-like epithelial cells (B and D).** FoxA2 significantly increased transcription in both cell types by acting on the 0.5-kb, 1.0-kb, and 2.0-kb proximal mouse *Cldn6* promoters ligated into luciferase reporter vectors. Gata-6 did not significantly increase *Cldn6* transcription in Beas2B cells transfected with the 0.5-kb construct **(C)**; however, Gata-6 significantly increased transcription in Beas2B cells when the 1.0-kb and 2.0-kb constructs were available and in all reporter experiments performed in A-549 cells **(D)**. Significant differences in luciferase activity normalized to β-galactosidase are noted at *P ≤ 0.05 when compared to non-transfected cells. The data shown are representative of experiments performed in triplicate.

Due to the observation that Cldn6 was detected in TTF-1 expressing pulmonary epithelial cells as well as in cells that express FoxA2, one of its known transcriptional partners, we determined whether these factors directly influenced the transcription of the *Cldn6* gene. Direct regulation by transcription factors was assessed in Beas2B (human Bronchiolar epithelial cells) and A-549 cells (an immortalized cell line characteristic of alveolar type II cells). Our data supported the concept that TTF-1 transcriptionally upregulated luciferase reporter plasmids that contained 0.5, 1.0, or 2.0-kb of the *Cldn6* promoter (Figure 5). Transcription of *Cldn6* mediated by TTF-1 was observed in both proximal Beas2B cells and distal A-549 cells. These experiments were repeated with expression vectors for FoxA2 and Gata-6. Gata-6 is a zinc finger containing transcription factor that like FoxA2, is expressed by respiratory epithelial cells where it plays a critical role in endoderm formation [35]. Gata-6 is a genetic target of TTF-1 that is essential in the viability of bronchiolar epithelial cells during morphogenesis [35] and is also a central player in alveologenesis and secondary septation of the immature alveolus [36]. Our data demonstrated that FoxA2 was sufficient to upregulate the three reporter plasmids containing increasing lengths of the *Cldn6* promoter (Figure 6A and B). Furthermore, Gata-6 was also effective in transcriptionally elevating *Cldn6* expression in both Beas2B and A-549 cells (Figure 6C and D). However, *Cldn6* transcription was only increased by Gata-6 in the 1.0 and 2.0-kb reporters and Gata-6 did not significantly activate the reporter that contained the 0.5-kb *Cldn6* promoter (Figure 6C).

## Discussion and conclusions

### Cldn6 expression in the developing lung

Cldns have dynamic, multimodal patterns of expression that for some family members, commence at the earliest stages of mammalian embryogenesis. An example of functional Cldn6 at the commencement of embryogenesis is observed when it cooperates with Cldn4 in the stabilization of trophectoderm located at the periphery of the blastocyst [37]. In fact, removal of Cldn6 and Cldn4 from the trophectoderm elicited hydostatic pressure imbalances that caused an arrest in development due to a collapse of the blastocyst.

The current research demonstrated that Cldn6 is expressed by developing respiratory epithelium at very early periods of morphogenesis and that its apical and basolateral expression is precisely controlled. Our discoveries relating to the notably high midgestational expression of Cldn6 and its marked decrease as development continues illustrates the notion that Cldn6 functions in the early programming stages of lung development. This concept is supported by previous research that revealed sporadic peaks in the expression of Cldns in diverse tissues during organogenesis followed by periods of diminished expression. For example, Cldns are associated with brain ventricle morphogenesis, particularly in relation to the derivation of the blood brain barrier [38]. Lei *et al.* demonstrated that cell adhesion proteins specific to the developing intestine recruit Cldn family members necessary for the initial formation of the intestinal barrier before their expression detectibly decreases [39]. Westmoreland *et al.* discovered that Cldn6, and to a lesser degree Cldn4 and Cldn12, were each highly expressed in the developing pancreas that like the lung, undergoes a programmed set of branching events during morphogenesis [40]. Their research revealed a distinctive, dynamic distribution pattern of Cldns6, 4, and 12 that related to elevated expression during pancreatic morphogenesis and altered expression during neoplastic disease [40]. Lastly, a theme of augmented Cldn expression during organogenesis and tapered expression following organ formation was detailed in research that centered on nephrogenesis [41]. Research revealed that nephric ducts, ureteric buds, and their derivatives robustly expressed Cldn3 during renal tubule formation and branching. Even though Cldn3 expression normally diminishes following organogenesis, the reintroduction of Cldn3 constructs caused *de novo* tubule branching to occur [41]. Our observation that Cldn6 is specifically expressed by apical and basolateral areas of branching pulmonary epithelial cells suggests roles central to their development. Because distinct boundaries of Cldn expression are observed in sites that correspond to inductive interactions during embryogenesis, further research may clarify whether Cldns are required in the translation of external signals into morphogenetic outcomes.

Our discovery that Cldn6 was co-expressed with Sox2 and Sox9 suggested plausible roles in the fate determination of developing airway and respiratory epithelium. Sox2 influences proximal airway epithelial cell differentiation and it has been recently implicated in canonical Wnt-β-catenin signaling [42]. Gain-of-function experiments showed that ectopically activated Wnt signaling negatively regulated Sox2 signaling required for bronchiolar lineage determination [31]. While additional research that seeks to identify links between Cldn6 and Sox2 is needed, the basis for such a link has been established by studies that show Wnt signaling orchestrates Cldn-mediated branching morphogenesis and angiogenesis [43]. While Sox9 is not essential for distal epithelial cell expansion and differentiation, it is considered a common marker for distal cell commitment. In addition to delineating such commitment, Sox9 cooperates with a host of other factors in the fine-tuning of distal cell phenotypes [44].

### Transcriptional control of Cldn6

Early in lung development, Cldn6 was expressed in the primordial tubules at sites also expressing TTF-1 [45]. TTF-1 regulates cytodifferentiation and formation of the respiratory epithelium [46]. Later in development (E13.5–E15.5), TTF-1, Gata-6, and FoxA2 are co-expressed by differentiating pulmonary epithelium [47,48]. The transcription factors TTF-1, Gata-6, and FoxA2 also significantly influence the transcription of other genes critical to lung function, including Clara Cell Secretory Protein (CCSP), and surfactant protein (SP)-A, SP-B, and SP-C. Our data revealed that *Cldn6* is also a transcriptional target of TTF-1, Gata-6, and FoxA2; therefore, the functions of *Cldn6* in lung organogenesis may relate to fundamental processes including cell population expansion, differentiation, and function. Furthermore, because TTF-1 regulates target gene expression in concert with other regulatory factors including CBP, PAX8, NFAT, NF-1, RAR, and AP-1, it is likely that the temporal-spatial distribution of Cldn6 expression is influenced in a highly complex fashion [44].

### Conclusions

The present study revealed that Cldn6 is both temporally and spatially controlled in the developing lung and that its regulation is maintained by critical transcriptional control networks managed by TTF-1. While Cldns are central to the coordination of barrier function and signaling, many questions specific to the roles of Cldn6 in the developing lung remain. Further studies are necessary to address uncertainties such as lung-specific redundancies, possible functions of Cldns in tissues that lack TJs, and whether these proteins have a future as therapeutic targets. Conditional gain-of-function and loss-of-function experiments in animal models may prove to be the most beneficial in deciphering the impact of Cldns on organ formation and maintenance.

## Competing interests

The authors declare that they have no competing interests.

## Authors’ contributions

FRJ, JBL, and TTW performed immunofluorescence and JBL and TTW completed the quantitative RT-PCR. FRJ and STB were responsible for the cell culture and reporter analyses. PRR conceived of the studies and with the assistance of FRJ, supervised in the implementation, interpretation, and writing. All authors assisted in manuscript preparation and approved of the final submitted version.
